# Vascularite cérébrale à *Mycobacterium tuberculosis*: à propos d’un cas pédiatrique

**DOI:** 10.11604/pamj.2025.52.171.49891

**Published:** 2025-12-18

**Authors:** Guylaine Aka Bossoma, Yves Constant Broh N'guessan, Jean Marc Konan Kouadio

**Affiliations:** 1 Université Félix Houphouët-Boigny de Cocody, Cocody, Abidjan, Côte d’Ivoire,; 2 Département de la Santé, de la Mère et de l'Enfant, Service de Pédiatrie, Centre Hospitalier et Universitaire, Treichville, Côte d'Ivoire,; 3 Département de Médecine et Spécialités, Service de Neurologie, Centre Hospitalier et Universitaire, Yopougon, Abidjan, Côte d'Ivoire

**Keywords:** Vascularite cérébrale, enfant, tuberculose, cas clinique, Cerebral vasculitis, child, tuberculosis, case report

## Abstract

La tuberculose du système nerveux cérébral est une urgence diagnostique et thérapeutique, source de décès et de séquelles parfois invalidantes. Elle peut se compliquer d'un accident vasculaire cérébral en cas de vascularite. Nous rapportons un cas pédiatrique. Nous rapportons le cas d'un enfant de 13 ans venu consulter pour un trouble de la conscience d'installation brutale en contexte fébrile. On note depuis un an des adénopathies cervicales non explorées et une toux. À J4 d'hospitalisation, il présente un syndrome pyramidal droit. L'imagerie par résonance magnétique cérébrale révèle un infarctus sylvien profond. La cytoponction ganglionnaire met en évidence le follicule de Koster. Le patient est mis sous traitement antituberculeux avec une évolution favorable.

## Abstract (en)

Tuberculosis of the cerebral nervous system is a diagnostic and therapeutic emergency that can lead to death and sometimes disabling sequelae. It can be complicated by stroke in the context of vasculitis. We report a pediatric case. We report the case of a 13-year-old child who presented with a sudden onset of altered consciousness in the context of fever. He had been experiencing unexamined cervical lymphadenopathy and a cough for a year. On day 4 of hospitalisation, he developed a right pyramidal syndrome. Cerebral magnetic resonance imaging revealed a deep middle cerebral artery infarction. Fine-needle aspiration of lymph nodes revealed Koster's follicle. The patient was started on antituberculosis treatment and achieved a favorable outcome.

## Introduction

Les vascularites cérébrales sont un groupe hétérogène de pathologies en rapport avec une inflammation diffuse des petites, moyennes ou grosses artères du cerveau et/ou de la moelle épinière. Elles peuvent être d'origine primitive ou secondaire. Le diagnostic positif des vascularites est difficile en raison de leurs étiologies diverses et de l'hétérogénéité des atteintes responsables de manifestations non spécifiques. Les étiologies infectieuses prédominent dans les vascularites secondaires, notamment la tuberculose. Nous rapportons le cas d'un adolescent immunocompétent traité pour une tuberculose multifocale compliquée d'une vascularite cérébrale.

## Patient et observation

**Information du patient:** K.E est un adolescent de 13 ans accompagné par ses parents pour un trouble de la conscience d'installation brutale et une fièvre. C'est un élève avec un bon développement psychomoteur. Il a reçu le vaccin contre la tuberculose et garde la cicatrice. Il vit dans une famille aux conditions socioéconomiques précaires, avec une promiscuité (maison d'une pièce pour neuf personnes). On ne note pas de contagion tuberculeuse. Il est hospitalisé pour des signes évoluant depuis un an associant des adénopathies cervicales indolores de taille différente, d'abord unilatérales puis bilatérales sans signe d'appel oto-rhino-laryngologiste; une fièvre intermittente, une asthénie, une anorexie. Les parents consultent dans un centre de santé où un antibiotique à large spectre est prescrit sans succès. D'autres consultations et médications sont réalisées (antibiothérapie, antipalustre, pharmacopée africaine) sans amélioration des signes. L'évolution est marquée deux semaines avant son admission par une fièvre intermittente non chiffrée avec des vomissements postprandiaux précoces pour lesquels une automédication est faite. Face à la persistance des signes et à l'installation trois jours avant, de propos incohérents, suivis d'un trouble de la conscience, les parents consultent pour avis et prise en charge.

**Résultats cliniques:** l'examen à l'entrée retrouve un état général conservé, un score de Glasgow à 13, un syndrome infectieux avec une fièvre à 39°C, une tachycardie à 140 battements par minute, un placard d'adénopathies mobile, ferme de taille différente (6, 4, 2 cm) avec un centre ramolli ayant tendance à la fistulisation dans l'aire sous mentonnière à droite et les aires jugulo-carotidienne et spinale à gauche, de fins râles crépitants dans le champ pulmonaire droit. On ne retrouve pas de syndrome méningé ni de déficit moteur. Il pèse 32 kg pour 1,5 m de taille, ce qui donne un indice de masse corporelle de 14,22.

**Figure 1:**
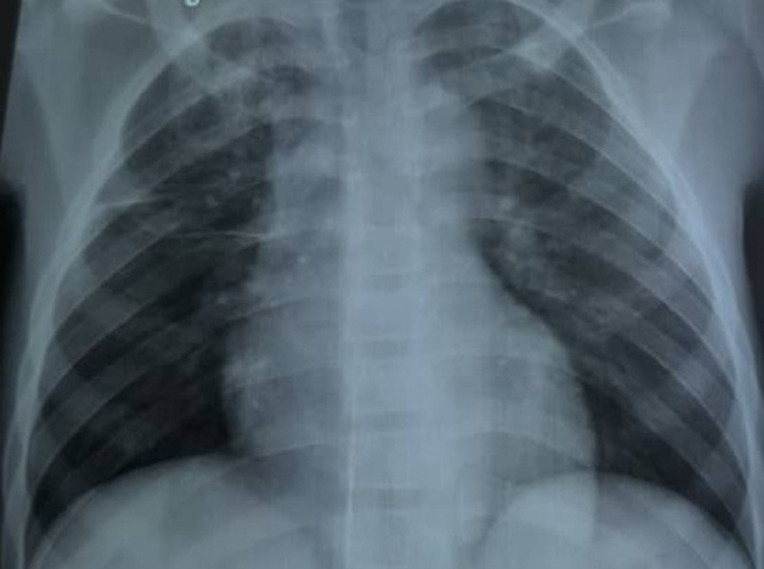
radiographique montrant des adénopathies hilaires et une excavation apicale à droite

**Démarche diagnostique:** le bilan biologique et radiologique retrouve: une discrète hyperleucocytose à polynucléaires neutrophiles (10 900/mm^3^ avec 88% PNN), une anémie hypochrome microcytaire à 7,5 g/dl, une protéine C réactive à 96 mg/l. Le scanner cérébral montre une absence de processus vasculaire, tumoral ou infectieux de l'encéphale; la radiographie thoracique montre une image cavitaire apicale droite avec un élargissement du médiastin (Figure 1).

**Figure 2:**
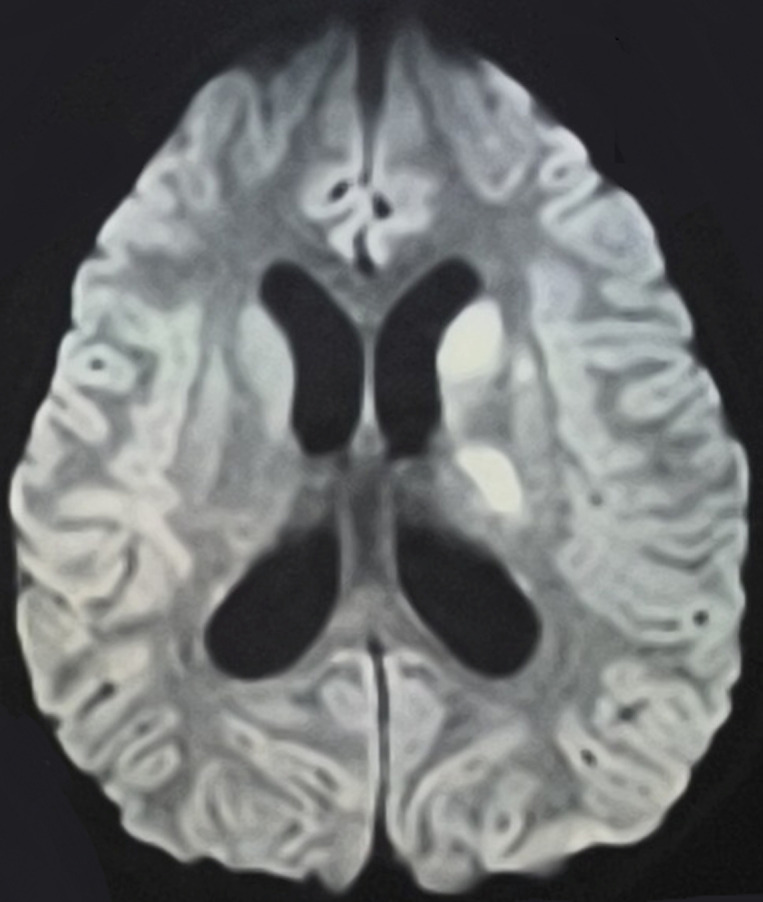
imagerie par résonance magnétique (IRM) d'un accident vasculaire cérébral ischémique

Le patient est traité pour une encéphalite bactérienne probable sous ceftriaxone injectable (100 mg/kg/j), aminoside injectable (5 mg/kg/j). À J4 d'hospitalisation, il présente une ptose palpébrale gauche, un syndrome pyramidal hémicorporel droit non proportionnel (membre inférieur droit 1/5; membre supérieur droit 3/5) avec persistance du syndrome infectieux et dégradation de l'état général, de l'état neurologique (score de Glasgow: 9-10). Une imagerie par résonance magnétique (IRM) cérébrale est demandée et objective un infarctus sylvien profond (noyaux gris bilatéraux prédominants à droite avec prise de contraste des méninges de la base faisant évoquer une méningo-vascularite (Figure 2)). Dans la recherche étiologique, l'intradermo-réaction à la tuberculine: anergie, une échographie abdominale: adénopathies profondes cœlio-mésentériques dont les plus importantes mesurent 13 et 5 mm de petit axe. Une cytoponction ganglionnaire est réalisée: adénite granulomateuse et nécrosante entrant dans le cadre d'une tuberculose au stade caséo-folliculaire. Le patient a été classé grade 2 par le *British Medical Research Council*. Le diagnostic retenu au terme de la démarche étiologique était une tuberculose multifocale compliquée d'un accident vasculaire cérébral, probablement d'une vascularite cérébrale. La glycémie veineuse à jeun, la sérologie du virus de l'immunodéficience humaine et l'électrophorèse de l'hémoglobine sont revenues à des valeurs normales.

**Intervention thérapeutique et suivi:** le patient a été mis sous antituberculeux (RHZE 2 comprimés/jour) et corticothérapie (solumédrol injectable pendant cinq jours puis relais par voie orale avec la prednisone pour une durée d'un mois). L'évolution a été marquée à J2 par une apyrexie avec amélioration progressive de l'état de conscience, à un mois, amélioration de l'état clinique avec néanmoins des séquelles motrices (aphasie, impotence fonctionnelle).

**Consentement du patient:** un consentement éclairé a été obtenu auprès des parents de l'enfant.

## Discussion

La tuberculose est une infection ancienne qui, malgré les stratégies de lutte, continue d'être un problème de santé dans le monde, en particulier dans les pays en développement d'Afrique, d'Asie et d'Amérique latine. En 2023, l'Organisation mondiale de la Santé note le taux le plus élevé depuis 1995: 8,2 millions de cas de tuberculose active [1]. Le germe se localise préférentiellement au niveau du parenchyme pulmonaire, mais des formes extrapulmonaires existent. L'atteinte du système nerveux central fait toute la gravité de la maladie avec une mortalité importante et le risque de survenue de séquelles souvent invalidantes. Elle représente 5 à 15% des cas de tuberculose extrapulmonaire [2]. Son évolution est insidieuse [3] et le diagnostic est difficile, en particulier chez l'enfant, devant: l'absence de spécificité clinique et la difficulté à isoler le germe, ce qui fait que le diagnostic est plus souvent présomptif [4]. Des complications peuvent survenir au cours ou à distance d'une méningite tuberculeuse. Elles sont de divers types: hydrocéphalie, accident vasculaire cérébral. L'accident vasculaire cérébral dans le cadre d'une tuberculose survient au décours d'une vascularite par une réaction immunologique [5]. Son incidence varie de 13 à 57% chez l'adulte. La vascularite des grosses artères se manifeste par des accidents vasculaires cérébraux avec syndrome pyramidal, dysarthrie, trouble de la conscience, comme ça a été le cas chez notre patient. Le diagnostic de vascularite est donné par l'histologie (biopsie vasculaire), mais il n'est pas réalisé en pratique courante. On s'appuie sur un faisceau d'arguments cliniques mais surtout radiologiques. L'imagerie cérébrale joue un rôle important, notamment l'angio-IRM ou l'IRM [6]. Ils permettent de visualiser des changements inflammatoires, d'étudier le parenchyme adjacent [7].

Le traitement de la vascularite repose sur le traitement étiologique. L'évolution est imprévisible, dépendant de la précocité du diagnostic, de l'observance thérapeutique, du terrain sur lequel elle survient.

## Conclusion

La tuberculose du système nerveux central peut se compliquer de vascularite responsable d'un accident vasculaire cérébral. Le diagnostic positif est difficile. Il repose sur un faisceau d'arguments cliniques, mais surtout sur l'imagerie cérébrale.
